# The Relationship between Internet Use and Self-Rated Health among Older Adults in China: The Mediating Role of Social Support

**DOI:** 10.3390/ijerph192214785

**Published:** 2022-11-10

**Authors:** Ningning Liu, Yujing He, Zhirong Li

**Affiliations:** 1 School of Public Administration, Central South University, Changsha 410083, China; 2 School of Architecture and Art, Central South University, Changsha 410083, China

**Keywords:** Internet use, older adults, self-rated health

## Abstract

The development of Internet technology has significantly impacted how people live their daily lives. How this disparity in Internet use affects the self-rated health of older adults needs to be further explored. This paper studies the impact of Internet use on self-rated health, aiming to examine the effect of Internet use and social support on the self-rated health of older adults in China. This study used data from the 2017 China General Social Survey (CGSS) to verify the effect of Internet use on older adults’ self-rated health. The results showed that Chinese elderly who used the Internet had a higher self-rated health, and social support from relatives and friends significantly improved the elderly’s self-rated health. This social support played a critical, partially mediating role between Internet use and self-rated health. In China, the effect of Internet use on older adults’ self-rated health was heterogeneous. Among them, the impact of Internet use was more significant for the male elderly, younger elderly, and rural elderly. The results suggest that the government should formulate targeted policies to improve the utilization of the Internet and maintain a good Internet environment to enhance the well-being of older adults according to the trend of population aging and the popularity of the Internet.

## 1. Introduction

Since the 1990s, China’s aging process has accelerated, showing rapid speed and large-scale characteristics. By 2020, China’s elderly population reached 190.64 million, accounting for 13.5% of the total population [1]. In the context of the development and popularity of Internet technology, we need to consider further the impact of Internet technology on the health of older adults. This reason is that difficulties in Internet use among older adults will lead to the health consequences of digital exclusion in the form of weakened social relationships and the deteriorating health status of older adults [2]. As of December 2021, the number of elderly Internet users aged 60 years or older in China reached 119 million, and the Internet penetration rate reached 43.2% [3]. Through these data, we have learned that there are significant differences in Internet use among the Chinese elderly population, which reflects a digital divide among the elderly. Today, with the development of China’s economy and technology, the access gap caused by differences in internet infrastructure has been bridged; however, the use gap based on the difference of digital technology applications has become the main form of a digital divide in China [4]. The digital use divide is particularly pronounced in older age groups. The causes of this digital divide can be found in the life course of older persons. China’s elderly groups have experienced rapid economic and technological development. Due to the impact of their original lifestyle, the elderly groups in the skills of learning appear vulnerable. Based on the popularity of Internet access and pcs in China, Zhao defines people born before 1975 as digital migrants who find it more difficult to adapting to the digital age [5]. As older digital migrants are less skilled, they will face a more difficult learning process. The digital anxieties felt by older groups will make them more psychologically resistant to internet use [6], leading to differences in Internet use among older groups. Therefore, based on the background of population aging and the digital divide among Chinese older adults, it is relevant to study the impact of Internet use on the physical health of Chinese older adults.

Studies by some scholars point out that Internet use has a negative effect on health. Studies have shown that online behavior compresses face-to-face communication between groups and thus affects the maintenance of interpersonal relationships [7], which leads to self-imposed isolation, inhibits the expression of personal emotions [8], and increases the tendency of Internet users to suffer from depression [9]. In addition, the Internet has broadened the information access platform for older adults, thus expanding their social comparison group. This comparison process with others, especially with upper social groups, creates a sense of relative deprivation in older adults, affecting their psychological feelings [10,11]. For the sake of clarity, health encompasses both mental and physical health. The above research shows the negative impact of Internet use on the health of the elderly, mainly reflected at the psychological level, such as depression, loneliness, and anxiety. However, we should not ignore the negative effects of Internet use on physical health and the effect of mental state on physical health. These negative effects are because there is a reciprocal relationship between mental and physical health; better mental health significantly impacts subsequent physical health [12,13].

In contrast, more research has shown that Internet use positively affects the self-rated health of older people [14,15,16,17]. Internet technology breaks the limitation of time and space and provides abundant information resources [18], which makes it possible for the elderly to obtain health information through online media. Health knowledge, information, resources, and services available to older adults through the Internet can effectively improve the health status of older users [19,20]. The Internet provides a platform for information sharing and doctor-patient interaction [21,22]. This online platform allows users to influence their health behaviors through information sharing and learning [22,23]. By providing information resources and establishing interactive communication platforms [23], the Internet has become an effective means of enhancing self-health management, accessing health information, and preventing disease [24,25].

Social support networks mediate the relationship between Internet use and self-rated health [17]. Studies on the relationship between the Internet and social support point out that the Internet provides access to social connections, which helps to increase the frequency of individuals’ social interactions [26,27]. The Internet offers new ways to connect with family, friends, homogeneous groups, and society [28,29]. It strengthens Internet users’ existing social networks in communication [30]. The Internet has effectively increased older adults’ social support and participation [9,31,32]. Social support means getting emotional encouragement, information, and help [33]. Adequate emotional support, information, and help during communication activities with family and friends can enhance older adults’ well-being and suppress negative emotions, thus contributing to the improved physical health of older adults [9,34].

In summary, studies remain divided on whether Internet use positively impacts older adults’ health. In addition, some studies have verified that social support can improve older adults’ health perceptions. Still, there is a lack of studies examining the relationship between Internet use and the self-rated health of older adults using social support as critical mediating variables. Therefore, this paper focuses on the differences in the health status of the elderly in China under the digital divide and discusses the influence of Internet use on the self-rated health of the elderly and the mediating effect of social support. The propensity score matching (PSM) method was used to solve the sample selection bias problem, which is easily ignored in previous studies. In addition, in mechanism analysis, more studies used comprehensive or single mediation variables without analyzing their inherent differences. Therefore, we use social support from relatives, friends, and neighbors as critical mediating variables. We can answer these questions by using the Karlson-Holm-Breen (KHB) method. (1) Does social support mediate the mechanism of Internet use’s impact on the elderly’s self-rated health? (2) The contribution of indirect effects for each mediating variable. (3) The contribution of the indirect effects of the single mediating variable.

## 2. Methods

### 2.1. Data Sources

We used data from the 2017 Chinese General Social Survey (CGSS). It uses stratified four-stage unequal probability sampling to comprehensively collect data at multiple levels: society, community, household, and individual. A total of 12,582 valid samples were completed in the 2017 CGSS. The older adults we want to study are those over 60 years old; therefore, we excluded the sample of people under 60. In addition, we also deleted the primary variable missing samples and non-logical samples. The final sample size was obtained at 4234.

### 2.2. Variable Selection

#### 2.2.1. Dependent Variable

The dependent variable is self-rated health. Self-rated health (SRH) is a critical assessment tool for aging and public health [35]. SRH was significantly associated with mortality, objective health indicators, and mental health [17,36]. When SRH is used to predict the general health of older adults, it is considered reliable and valid [37]. The CGSS questionnaire, “How do you feel about your current physical health?” is often used to measure respondents’ self-rated health [38,39]. This questionnaire question reflected objective health maintenance and subjective health perception. The survey divided the health level into five categories in a hierarchical order. The five categories were “very unhealthy”, “relatively unhealthy”, “average”, “relatively healthy”, and “very healthy”. The five categories of responses were sequentially assigned to a scale of 1–5 to measure the self-rated health status of the elderly population.

#### 2.2.2. Independent Variable

The independent variable is Internet use. Internet use by the elderly is whether the elderly population accesses Internet services through computers, cell phones, and other devices for communication, entertainment, health consultation, and other activities. This variable is measured by the CGSS questionnaire “In the past year, how much did you use the Internet (including cell phone access)?” The answer “never” means respondents have never been on the Internet, which is assigned as 0, and the other answers mean respondents use the Internet, which is set as 1.

#### 2.2.3. Mediating Variables

The Mediating variable is social support. As for social support, this paper extracts questions related to close contact, friend socialization, and neighborhood socialization from the CGSS questionnaire. We will use the questions in the questionnaire: “In the past year, have you often gathered with relatives who do not live together in your spare time?”, “How often do you socialize with other friends?”, “How often do you socialize with your neighbors?” The respondents’ answers to these three questions reflect the social support of the elderly group from relatives, friends, and neighbors.

#### 2.2.4. Control Variables

To avoid the endogenous problems caused by missing variables, we need to control the variables that may affect the self-rated health of older adults. It has been found that individual characteristics, family size, and social insurance can affect self-rated health [40,41]. Therefore, we will control these variables. The individual characteristics variables include gender, age, and education level. Household size variables include marital status, household size, and number of children. The social security variables include whether or not they participate in pension insurance and whether or not they participate in medical insurance. Our focus is on the health of older people in China, so we also need to control for individual social identity variables with Chinese characteristics, which include household registration and party membership. The household registration variable is controlled because China’s historical urban-rural dualistic structure has led to differences between urban and rural in terms of social security and the health status of older age groups [42]. In China, party membership is an important social identity that has an impact on its own utility [43]. Therefore, we also control for this variable.

### 2.3. Statistical Analysis

This paper focuses on the impact of Internet use on the self-rated health of older adults, and the study includes the following five main aspects:

First, a descriptive statistical analysis was performed. Second, the impact of Internet use on the self-rated health of older adults was analyzed. The self-rated health of older adults is a multinomial ordered selection variable, so a multivariate ordered probit regression model is used as the baseline regression model. Third, considering the possible selective bias between Internet use and older adults’ self-rated health, that is, older adults’ Internet use behavior is a non-random behavior influenced by individual traits of older adults. Since whether or not to use the Internet as a dichotomous variable, to control the possible selective bias, we used the propensity score matching (PSM) method to measure the net utility of Internet use on the self-rated health status of the elderly. Fourth, the mediating effect of social support between Internet use and older adults’ self-rated health was examined. We refer to the existing studies using the mediating effect causal step method and the Karlson, Holm, and Breen (KHB) mediation analysis method to test the mediating effect. Fifth, a subsample analysis was conducted to assess the impact of Internet use on self-rated health across different groups. All data were analyzed using STATA 17.0; two-tailed *p*-values less than or equal to 0.1 were considered statistically significant.

## 3. Results

### 3.1. Descriptive Analysis

Table 1 shows the descriptive statistics for the study variables. The mean self-rated health score was 2.977, according to the 1–5 standard of self-rated health evaluation, which means that most older adults think their physical condition is at the general level. 23.1% of the elderly said they use the internet, and 22.1% said they use it in their leisure time. The results show significant differences in Internet use among older people, and more older people do not use the internet. The average scores for socializing with relatives, friends, and neighbors were 2.104, 3.544, and 4.164, respectively. In contrast, the elderly have closer contact with their neighbors. The proportion of men and women in the study group was similar, with an average age of 69.341. The average number of children and family size were 2.422 and 2.455. However, the lowest and highest values of the number of children and family sizes differ significantly. Most of the subjects were married, and 71.2% were married and had spouses. Most elderly are covered by medical insurance (92.8%) and pension insurance (80.5%).

### 3.2. Baseline Regression Results Analysis

Table 2 shows the ordinal probit regression analysis results of the influence of Internet use on the self-rated health of the elderly.

Model 1 showed that the regression coefficient of Internet use on older adults’ self-rated health was positive and significant at the 1% level. In Model 2, we controlled the personal characteristics variable of older adults, and the results showed that the effect of Internet use on older adults’ self-rated health was still significant at the 1% level. In Model 3, we controlled the household size and social security variables of older adults, and the results showed that the effect of Internet use on older adults’ self-rated health remained significant at the 1% level. The above results imply that Internet use significantly affects the self-rated health of the elderly population, which means Internet use can improve the health of the elderly population. Regarding control variables, Men, Party members, and people with high education levels have a higher self-rated health levels. There was a negative relationship between the number of children and the level of self-rated health.

Models 4 and 5 show the results of the robustness tests. We test the robustness by replacing models and variables. Model 4 shows the regression results of replacing the ordered Probit model with the ordered Logit model. The results show that Internet use positively affects the self-rated health of older adults and is significant at the 1% level. In Model 5, the leisure time Internet use variable was used as a proxy variable for Internet use. This variable was derived from the question “In the past year, did you regularly engage in online activities in your leisure time?” which was obtained from the question with a value of 1 indicating Internet access in leisure time and 0 indicating no Internet access in leisure time. From model 5, it is clear that Internet access in leisure time has a significant positive effect on self-rated health.

We should note that the regression coefficients in Table 2 reflect only their effectiveness on self-rated health, not the marginal effects. Therefore, we further investigated the marginal effect of Internet use on the self-rated health of the elderly. The results are shown in Table 3. Compared with older people who did not use the internet, older people who did use the internet had a 2.79 percent lower chance of being “Very unhealthy” and a 3.88 percent lower chance of being “Relatively unhealthy”. The probabilities of being “Relatively healthy” and “Very healthy” would rise by 4.19 percent and 2.40 percent, respectively.

### 3.3. Robustness Test

The effect of Internet use on self-rated health was investigated above. Although we have previously tested robustness by substituting variables and models, the problem of selection bias in the sample of elderly respondents has not been solved. This paper uses k-nearest neighbor matching, radius neighbor matching, and kernel matching to estimate. To ensure the matching effect, we need to test the equilibrium of sample matching quality. We conducted a balance test on the PSM results to observe whether there was a significant difference in the distribution of matching variables between the matched samples. As shown in Table 4, the proportion of bias for each variable after matching was less than 5%. There was no significant difference between the treatment group (using the Internet) and the control group (not using the Internet), indicating that the samples’ selective bias can be eliminated largely by propensity score matching.

**Figure 1:**
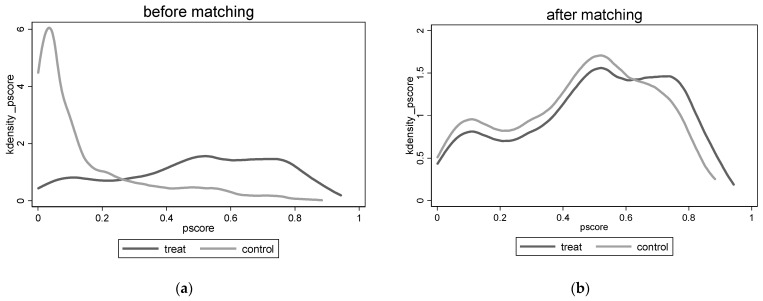
Kernel density maps: (**a**) before matching; (**b**) after matching.

In this paper, we also report the kernel density map before and after matching, as shown in Figure 1. After matching, the curves of the treatment and control groups overlap nicely, and the two curves show a more similar trend indicating that the particular bias problem of the samples was effectively solved by propensity score matching.

Table 5 shows the mean treatment effect (ATT) of Internet use on the self-rated health of older adults. Before matching, the mean treatment effect was 0.467, which was significant at the 1% level. In k-nearest neighbor matching, the mean treatment effect was 0.201, which was significant at the 1% level, indicating that after controlling for sample selectivity bias, the self-rated health levels of older adults who use the Internet are approximately 0.201 higher than those who do not use the Internet. The data were matched for accuracy using radius matching and kernel matching. In radius neighbor matching, the mean treatment effect was 0.188. In kernel matching, the mean treatment effect was 0.177. The mean treatment effects obtained from the matching were similar and significant at the 1% level, indicating that the results obtained from propensity score matching are reliable. It also suggests that without addressing the endogeneity issue, we would overestimate the positive impact of Internet use on the self-rated health of older adults.

### 3.4. Mechanism Analysis

**Figure 2:**
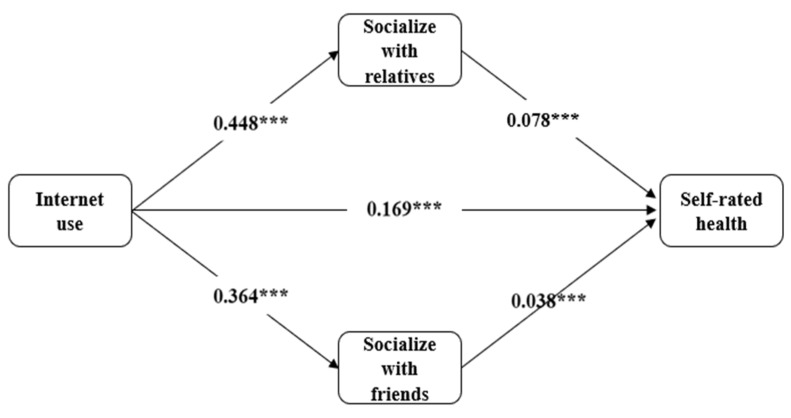
The Mediating Relationship Between Internet Use and Self-rated Health and the Non-standardized Regression Coefficients (Matched Samples): Standard errors in parentheses *** *p* < 0.01.

In this section, we will analyze the mechanisms by which Internet use affects older adults’ self-rated health under the mediating role of social support. We use a k-nearest neighbor-matched sample to test the mediating effect. We first refer to the idea of mediation effect analysis by Zhonglin Wen and Baojuan Ye to verify the mediation effect [44]. Table 6 shows the analysis results for the matched samples. The results show that Internet use positively affects the social networks of older adults’ relatives and friends, which is significant at the 1% level. However, the effect of Internet use on older adults’ neighborhood social interactions was insignificant. Therefore, we did not include neighborhood social variables in the last step of the regression. As shown in the previous column of Table 6, we added the variables of social interaction with relatives and social interaction with friends to Model 3. The coefficient and significance of the effect of Internet use on older adults’ self-rated health decreased with the inclusion of these two variables suggesting that these two variables mediate between Internet use and older adults’ self-rated health. Figure 2 shows a visualization of the mediating effect.

Because we used an ordered probit model with multiple mediating variables, we used the KHB method to test further the mediating effects of relatives, friends, and neighbors [45]. Table 7 shows the results of the KHB test for a single mediating variable. The direct impact of the independent variable (Internet use) decreases when socializing with relatives and socializing with friends are included separately, which indicates that socializing with relatives and socializing with friends have indirect effects, and such indirect effects are significant at the 1% level. The direct effect of the independent variable (Internet use) did not significantly decrease when the variable of socializing with neighbors was included, and the indirect effect of socializing with neighbors was not significant. The results of the KHB analysis were similar to the stepwise test for mediating effects described above, which further demonstrates that socializing with relatives and socializing with friends can be used as mediating variables to explain the effects of Internet use on older adults’ self-rated health, increasing credibility.

The above results validate that interaction with relatives and interaction with friends can be used as mediating variables to explain Internet use’s effect on older adults’ self-rated health. In this section, we elucidate the contribution of the indirect effects of the two mediating variables and the contribution of the indirect effects of the single mediating variable. We included two mediating variables, interaction with relatives and interaction with friends, and used the KHB method to calculate the contribution of indirect effects for each mediating variable. When all mediating variables were included, the two variables, socializing with relatives and socializing with friends, accounted for 32.26% of the overall model, with socializing with relatives accounting for the most significant proportion and 52.89% of the indirect effect.

### 3.5. Heterogeneity Analysis

With the above data results, we verified the impact of Internet use on the self-rated health of older adults and how Internet use affects the self-rated health of older adults by influencing their interactions with family and friends. However, Internet use can have different effects on the self-rated health status of different groups of older adults. Therefore, in this section, we further investigate the heterogeneity of the effects of Internet use on the self-rated health of different groups of older adults. We analyze heterogeneity by gender, age, and household registration. To avoid the endogeneity problem, we still use the k-nearest neighbor matching method, radius matching method, and kernel matching method to analyze the effects of the Internet on different groups of older adults and use the sample after k-nearest neighbor matching to test the mediating effects. The results are shown in Table 8 and Table 9.

In the analysis of heterogeneity by gender, the mean treatment effect for men was 0.309, which was significant at the 1% level, while the mean treatment effect for women was insignificant.

For age differences, we divided the ages into a low age group of 70 years and younger and a high age group of 70 years and older. As shown in Table 8, before sample matching, the mean treatment effect for the lower age group was 0.210, which was significant at the 1% level. The mean treatment effect for the higher age group was insignificant.

Regarding household differences, we divided the overall sample into rural and non-rural samples. The results showed that the self-rated health status of older adults in the rural group increased by approximately 33.7%, and the mean treatment effect was significant at the 1% level. Older adults in the non-rural group had an increase in the self-rated health status of approximately 13.4%, and the mean treatment effect was significant at the 5% level.

These results indicate that Internet use did not significantly affect self-rated health in the female and older age groups. Therefore, in analyzing the mediating role of social support in the effect of Internet use on the self-rated health of older adults, we analyzed only the male group, the lower age group, the rural household group, and the non-rural household group. Table 9 shows the results of validating the mediating role of social support in the effect of Internet use on the health of older adults using the KHB method. The results show that the indirect effect of the socialization variable with neighbors was insignificant across the groups. The indirect effects of the variables socializing with relatives and friends were significant at the 1% level in the male, non-rural and lower age samples. On the contrary, the indirect effects of the variables of interaction with relatives and interaction with friends were not significant in the rural sample.

## 4. Discussion

The results of this study showed that Internet use had a significant positive impact on the self-rated health of older Chinese adults. This finding is consistent with many previous related studies indicating that Internet use positively impacts the self-rated health of older Chinese adults. The Internet provides many health information and medical resources [46], allowing older adults to maintain their objective health more conveniently. Convenient and effective access to health advice and information allows older adults to access high-quality health services and reliable health diagnostic information across space, influencing their healthy lifestyles [47].

The Internet can increase the frequency of interaction between older adults and their relatives and friends. The Internet has provided a convenient and effective communication channel for the elderly in China and strengthened their social contacts. In social contact, support from family and friends was positively correlated with self-rated health [48]. Our study supports this view. The results of data analysis showed that interactions with family and friends significantly improved older adults’ self-rated health, suggesting that social support from family and friends plays a crucial mediating role in the effect of Internet use on older adults’ self-rated health suggesting that older adults can not only use the Internet to improve their health but also enhance their interactions with family and friends through the Internet and use more social support resources to improve their health and cognitive status.

Internet use had different effects on the self-rated health of older adults by gender and age group. Internet use positively and significantly affected self-rated health for older men. Still, Internet use did not considerably affect self-rated health for older women. Gender differences in the health effects of the Internet can be supported by previous studies. Previous studies have shown that Internet use has more obvious health effects on males [49]. There are differences between men and women in Internet behavior. Among them, men are more likely to participate in informational and instrumental Internet activities [50], and men show more online health information-seeking behavior [51]. For older adults in different age groups, Internet use improved self-rated health in the younger group but had no significant effect on self-rated health in the older group. In terms of age, one study analyzed the impact of Internet use on the middle-aged and older age groups, noting that the middle-aged group used the Internet more and had more accessible access to information to improve their health [52]. Thus, compared to the elderly population, the younger group has a relatively higher awareness and use of the Internet, thus improving their health.

Internet use significantly impacts the self-rated health of urban and rural elderly populations. Earlier studies using data from the 2013 China Integrated Social Survey demonstrated that Internet use improves the physical and mental health of urban older adults in China but has only a positive effect on the physical health of rural older adults in China [53]. Lin X. also verified that Internet use significantly affects the mental health of urban older adults using data from the 2014 and 2016 China Digital Ageing Social Survey (CLASS), but no significant effect on the mental health of rural older adults. He also attributed the difference between urban and rural areas to the development of the urban-rural dichotomy, lower Internet use in rural areas, and lower educational attainment of rural residents [9]. In contrast to these findings, our study found that the effect of Internet use on self-rated health was more significant among rural residents than urban residents. Previous studies have found that the Information and Communication Technology (ICT) channel of health information acquisition has a more positive effect on rural residents’ health literacy cultivation than urban residents [54]. The differences in e-health literacy among the elderly will affect health outcomes [55]. Therefore, the expansion of electronic information channels can explain the impact of Internet use on the self-rated health of older people in urban and rural areas. Currently, the digital access gap between urban and rural areas in China is narrowing; the development of mobile Internet technology has reduced the cost of using the Internet for rural residents; thus, rural residents can obtain information elements spanning time and space [56]. In 2017, the Shanghai University of Finance and Economics focused on the application of the Internet in rural areas in 31 provinces, municipalities, and autonomous regions of China (excluding Hong Kong, Macao, and Taiwan) and conducted a survey. According to the results of 10,381 questionnaires collected, 92.98% of households in rural areas have cell phones, the Internet penetration rate of rural households is 62.19%, and 4G networks have covered 88.43% of surveyed villages [57] indicated that the Internet user penetration rate in rural areas has improved, and Internet activities have become essential to rural residents’ lives.

We analyzed the mediating role of social support on groups affected by Internet use. The results showed that social support did not mediate the effect of Internet use on self-rated health in the rural elderly group, unlike the male group, the younger elderly group, and the urban elderly group. There is a complex relationship between regional context and social interactions of older adults [58]. Regional differences in social interaction patterns will substantially impact older adults’ health [59]. This paper verified regional differences in the impact of social interaction patterns on the health of older adults. The differences in social contact patterns between urban and rural areas are manifested in the acquaintance society in rural areas and the stranger society in urban areas. Among them, the higher homogeneity and closer communication of rural social groups and the higher heterogeneity of urban communities make urban older adults focus more on contact with friends and relatives; therefore, social support from friends and relatives can alleviate social isolation and improve self-rated health.

This paper also had some limitations. We used data from the 2017 China General Social Survey, the latest data published by the current China General Social Survey. The data used in this study date from 2017, well before the Corona pandemic. The COVID-19 pandemic has restricted people’s travel, especially those who have been medically quarantined, and it is difficult for them to have contact with other people. In this case, the Internet has become an essential way of communication, information transmission, and health consultation. The Internet involves travel, medical care, and communication, so the elderly are required to use the Internet for technical support. Therefore, the occurrence of COVID-19 has seriously affected the health information and social interaction behavior of the elderly using the Internet, bringing new problems and discoveries. Yet, this article cannot discuss these possible phenomena due to data limitations. However, this provides a direction for future research. We will compare pre-and post-pandemic internet use with self-rated health in older adults when new data become available in the future.

## 5. Conclusions

In summary, based on data from the 2017 China General Social Survey, we used an ordered probit model to explore the effect of Internet use on the self-rated health of older Chinese adults. To improve the robustness of the results, we tested the robustness through variable and model changes. In addition, because the Internet use behavior of older adults is a non-random behavior influenced by the characteristics of older adults, the PSM method is used in this paper to address the problem caused by sample selection bias. Afterward, the internal mechanism of Internet use affecting older adults’ self-rated health was analyzed using matched samples. The mediating effect of social support from family and friends between Internet use and older adults’ self-rated health was investigated using the mediating effect causal step method and the KHB method. Considering the effects of Internet use on the self-rated health of different elderly groups, we further analyzed the heterogeneity by gender, age, and household registration. The results showed that Internet use significantly affected older adults’ self-rated health. Social support from family and friends partially mediated the effect between Internet use and self-rated health among older Chinese adults. However, there is a group heterogeneity in this result. Specifically, Internet use has a significant impact on the self-rated health of the male elderly group and the younger age group but not on the female elderly group and the older age group. Although Internet use significantly impacts the urban and rural elderly population’s self-rated health, the impact of Internet use on the physical self-rated health of the rural elderly is more significant. The mediating effect of social support is essential in the male elderly, young, elderly, and urban elderly groups, but not in the rural elderly group, which is related to the social interaction model in rural areas.

The findings of this study have several important policy implications.

First, we should accelerate the popularization of Internet applications, mainly to narrow the digital gap between urban and rural areas and to increase the utilization of the Internet by the elderly. The Internet is a health management platform, information communication platform, and leisure and entertainment platform that older adults can effectively utilize. Differences in Internet usage opportunities among different elderly groups will lead to differences in healthy living, well-being, and access to support. Currently, China is promoting an urban-rural integration strategy through the rural revitalization strategy, and the quality of life in rural areas has been significantly improved. In this development process, it is necessary to promote the construction of rural network infrastructure, continuously deepen information services, and cultivate digital literacy among rural residents. In particular, developing information services and digital skills training for the elderly will enable the rural elderly to use the Internet better to obtain health information and social support and improve their sense of well-being.

Second, the government needs to cooperate with Internet enterprises to promote the transformation of the Internet to adapt to aging and provide Internet services suitable for the elderly. In 2021, China will carry out nationwide aging and accessibility actions for Internet applications and incorporate aging-appropriate evaluation into enterprise credit evaluation. The aging transformation of the Internet will help to address the barriers to Internet use by the elderly and increase the use of the Internet and its use by the elderly community. The simplicity and convenience of Internet use for the elderly will enable them to better access information and services, meet their information life needs, and improve their Internet experience.

Finally, the government should strengthen the regulation of virtual communities established through online media, screen excessive useless and harmful information, provide a safe online environment for the elderly, and prevent online fraud and information overload. The Internet is a double-edged sword. It provides a platform for healthy information delivery and social exchange and facilitates the spreading of false information and fraudulent information. With the development of the Internet, more and more information has appeared on online platforms, making it difficult for people to distinguish the reliability of the data. Under information asymmetry, older adults are highly vulnerable to the impact of false information on the Internet, which will seriously threaten their physical health, psychological health, and property security. Therefore, it is necessary to strengthen the supervision of online platforms to protect the rights and interests of the elderly.

## Figures and Tables

**Table 1:** Descriptive univariate information for variable.

Variables	Mean	SD	Min	Max
Self-rated health	2.977	1.077	1	5
Internet use	0.231	0.421	0	1
Leisure	0.221	0.415	0	1
Socialize with relatives	2.104	1.057	1	5
Socialize with friends	3.544	2.054	1	7
Socialize with neighbors	4.164	2.291	1	7
Gender	0.484	0.450	0	1
Age	69.341	7.377	60	103
Household register	0.483	0.450	0	1
Party member status	0.155	0.362	0	1
Education	6.652	4.619	0	19
Marriage	0.712	0.453	0	1
Children	2.422	1.405	0	12
Family size	2.455	1.500	1	23
Medical insurance	0.928	0.260	0	1
Endowment insurance	0.805	0.396	0	1

**Table 2:** Baseline regression results.

Variable	Model 1 (Oprobit)	Model 2 (Oprobit)	Model 3 (Oprobit)	Model 4 (Ologit)	Model 5 (Oprobit)
Internet use	0.455 ***	0.198 ***	0.186 ***	0.320 ***	
	−0.039	(0.0453)	(0.0454)	(0.0776)	
Leisure					0.194 ***
					(0.0459)
Gender		0.136 ***	0.127 ***	0.232 ***	0.129 ***
		(0.0339)	(0.0342)	(0.0588)	(0.0342)
Age		−0.00612 ***	−0.00247	−0.00319	−0.00252
		(0.00230)	(0.00264)	(0.00457)	(0.00264)
Household register		0.188 ***	0.160 ***	0.276 ***	0.161 ***
		(0.0399)	(0.0410)	(0.0711)	(0.0410)
Party member status		0.0832 *	0.0818 *	0.137	0.0767
		(0.0492)	(0.0492)	(0.0835)	(0.0493)
Education		0.0254 ***	0.0241 ***	0.0418 ***	0.0242 ***
		(0.00461)	(0.00465)	(0.00802)	(0.00463)
Marriage			0.0381	0.0529	0.0376
			(0.0389)	(0.0669)	(0.0389)
Children			−0.0375 ***	−0.0652 ***	−0.0367 ***
			(0.0136)	(0.0234)	(0.0136)
Family size			0.00538	0.00909	0.00528
			(0.0111)	(0.0189)	(0.0111)
Medical insurance			−0.0543	−0.0916	−0.0568
			(0.0655)	(0.114)	(0.0655)
Endowment insurance			0.0439	0.0984	0.194 ***
			(0.0433)	(0.0748)	(0.0459)
Observation	4234	4234	4234	4234	4234
Log-likelihood	−6128.9881	−6057.4164	−6052.3615	−6050.2103	−6051.8551
Pseudo R2	0.0113	0.0228	0.0237	0.0240	0.0237
LR chi2	140.00	283.15	293.26	297.56	294.27

Standard errors in parentheses *** *p* < 0.01, * *p* < 0.1.

**Table 3:** Marginal effects of Internet use on self-rated health of the elderly.

Variable	Y = 1	Y = 2	Y = 3	Y = 4	Y = 5
Self-rated health	Very unhealthy	Relatively unhealthy	General	Relatively healthy	Very healthy
Internet use	−0.0279 ***	−0.0388 ***	0.0008	0.0419 ***	0.0240 ***
	(0.0069)	(0.0095)	(0.0009)	(0.0102)	(0.0059)

Standard errors in parentheses *** *p* < 0.01.

**Table 4:** Covariates balance testing for propensity score matching.

Variables	Unmatched	Mean Value		Deviation %	Deviation Reduction Ratio %	*T*-Test	
	Matched	Treated	Control			*T*-Value	*p* > |t|
Gender	U	0.536	0.468	13.6		3.74	0.000
	M	0.535	0.538	−0.5	96.0	−0.12	0.905
Age	U	66.846	70.090	−46.8		−12.26	0.000
	M	66.904	66.845	0.9	98.2	0.21	0.837
Household register	U	0.854	0.372	113.9		28.94	0.000
	M	0.852	0.855	−0.7	99.4	−0.18	0.855
Party member status	U	0.304	0.110	49.3		15.10	0.000
	M	0.299	0.292	1.7	96.5	0.33	0.741
Education	U	10.584	5.473	130.9		34.29	0.000
	M	10.532	10.511	0.5	99.6	0.13	0.897
Marriage	U	0.802	0.684	27.3		7.19	0.000
	M	0.801	0.793	1.9	93.1	0.44	0.658
Children	U	1.658	2.651	−78.9		−20.29	0.000
	M	1.665	1.683	−1.4	98.3	−0.37	0.713
Family size	U	2.440	2.459	−1.3		−0.35	0.730
	M	2.415	2.442	−1.8	−39.2	−0.45	0.655
Medical insurance	U	0.958	0.918	16.5		4.21	0.000
	M	0.958	0.957	0.3	98.4	0.07	0.943
Endowment insurance	U	0.890	0.780	29.7		7.62	0.000
	M	0.888	0.892	−1.1	96.4	−0.28	0.780

**Table 5:** Propensity score matching (PSM) estimation for the effect of Internet use on self-rated health.

Matching Method	Treated	Controls	ATT	S.E.	T-Stat
Unmatched	3.335	2.869	0.467	0.039	12.09
Matched					
K-nearest neighbor (*n* = 4)	3.336	3.134	0.201 ***	0.060	3.35
Radius matching	3.336	3.148	0.188 ***	0.058	3.24
Kernel	3.336	3.159	0.177 ***	0.055	3.22

Standard errors in parentheses *** *p* < 0.01. The bootstrap method obtained standard errors after matching, and the number of self-help samples was 500. K-nearest neighbor matching adopts “one to four” matching. The radius of k-nearest neighbor caliper matching and radius adjacent matching is 0.01. The default values of kernel function and bandwidth are used in kernel matching.

**Table 6:** The mediating effect of social support.

	Self-Rated Health	Socialize with Relatives	Socialize with Friends	Socialize with Neighbor	Self-Rated Health
Internet use	0.169 ***	0.448 ***	0.364 ***	−0.001	0.116 **
	(0.050)	(0.051)	(0.049)	(0.050)	(0.051)
Socialize with relatives					0.078 ***
					(0.027)
Socialize with friends					0.038 ***
					(0.014)
Socialize with neighbor					
				
Control variables	YES	YES	YES	YES	YES
Observation	2041	2041	2041	2041	2041
Log-likelihood	−2889.4679	−2618.0525	−3848.9463	−3733.2475	−2874.5523
Pseudo R2	0.0156	0.0301	0.0099	0.0171	0.0207
LR chi2	91.87	162.42	77.28	130.08	121.71

Standard errors in parentheses *** *p* < 0.01, ** *p* < 0.05.

**Table 7:** Effect decomposition and comparison of KHB methods.

Mediating Variables	Dependent Variables: Self-Rated Health				
	Total Effect	Direct Effect	Indirect Effect	Proportion in Indirect Effect	Proportion in Total Effect
Socialize with relatives	0.170 ***	0.128 **	0.0423 ***	52.89%	17.06%
	(−0.050)	(0.050)	(0.010)		
Socialize with friends	0.170 ***	0.132 ***	0.0383 ***	47.11%	15.20%
	(0.050)	(0.050)	(0.010)		
Socialize with neighbors	0.169 ***	0.169 ***	0.001	-	-
	(0.050)	(0.050)	(0.004)		
Social support	0.171 ***	0.116 **	0.055 ***	-	32.26%
	(0.050)	(0.051)	(0.012)		

Standard errors in parentheses *** *p* < 0.01, ** *p* < 0.05.

**Table 8:** Results of heterogeneity analysis. (PSM Estimate).

Matching Method	Gender		Age		Household Register	
	Female	Male	Low	High	Rural	Non-Rural
K-nearest neighbor (*n* = 4)	−0.031	0.309 ***	0.210 ***	0.081	0.337 ***	0.134 **
	(0.089)	(0.081)	(0.074)	(0.091)	(0.118)	(0.068)
Radius matching	−0.025	0.339 ***	0.182 **	0.070	0.386 ***	0.144 **
	(0.085)	(0.076)	(0.073)	(0.090)	(0.110)	(0.064)
Kernel	−0.042	0.356 ***	0.210 ***	0.102	0.401 ***	0.140 **
	(0.081)	(0.073)	(0.069)	(0.084)	(0.106)	(0.061)

Standard errors in parentheses *** *p* < 0.01, ** *p* < 0.05.

**Table 9:** Results of heterogeneity analysis. (KHB Estimate).

Group	Independent Variable	Mediating Variables	Dependent Variables: Self-Rated Health		
			Total Effect	Direct Effect	Indirect Effect
Male (N = 1098)	Internet use	Socialize with relatives	0.347 ***	0.294 ***	0.0531 ***
			(0.0681)	(0.0688)	(0.0148)
		Socialize with friends	0.346 ***	0.291 ***	0.0548 ***
			(0.0681)	(0.0692)	(0.0157)
		Socialize with neighbors	0.344 ***	0.333 ***	0.0104
			(0.068)	(0.068)	(0.008)
Low age (N = 1349)	Internet use	Socialize with relatives	0.202 ***	0.155 **	0.0470 ***
			(0.0610)	(0.0622)	(0.0142)
		Socialize with friends	0.201 ***	0.166 ***	0.0349 ***
			(0.0610)	(0.0619)	(0.0123)
		Socialize with neighbors	0.201 ***	0.194 ***	0.00702
			(0.0610)	(0.0610)	(0.00601)
Rural (N = 544)	Internet use	Socialize with relatives	0.328 ***	0.311 ***	0.0172
			(0.104)	(0.105)	(0.0175)
		Socialize with friends	0.327 ***	0.325 ***	0.00157
			(0.104)	(0.106)	(0.0240)
		Socialize with neighbors	0.327 ***	0.317 ***	0.0103
			(0.104)	(0.104)	(0.0122)
Non-rural (N = 1484)	Internet use	Socialize with relatives	0.148 ***	0.0979 *	0.0499 ***
			(0.057)	(0.058)	(0.013)
		Socialize with friends	0.148 ***	0.106 *	0.0417 ***
			(0.057)	(0.058)	(0.0112)
		Socialize with neighbors	0.147 **	0.148 ***	−0.00159
			(0.057)	(0.057)	(0.004)

Standard errors in parentheses *** *p* < 0.01, ** *p* < 0.05, * *p* < 0.1.

## Data Availability

The data were released to the researchers without access to any personal data. Data access link: http://cgss.ruc.edu.cn/ (accessed on 27 November 2020).
